# Crucial Residue Involved in L-Lactate Recognition by Human Monocarboxylate Transporter 4 (hMCT4)

**DOI:** 10.1371/journal.pone.0067690

**Published:** 2013-07-31

**Authors:** Shotaro Sasaki, Masaki Kobayashi, Yuya Futagi, Jiro Ogura, Hiroaki Yamaguchi, Natsuko Takahashi, Ken Iseki

**Affiliations:** 1 Laboratory of Clinical Pharmaceutics and Therapeutics, Division of Pharmasciences, Faculty of Pharmaceutical Sciences, Hokkaido University, Sapporo, Japan; 2 Hokkaido Pharmaceutical University School of Pharmacy, Otaru, Japan; 3 Department of Pharmacy, Hokkaido University Hospital, Sapporo, Japan; University of Cambridge, United Kingdom

## Abstract

**Background:**

Monocarboxylate transporters (MCTs) transport monocarboxylates such as lactate, pyruvate and ketone bodies. These transporters are very attractive therapeutic targets in cancer. Elucidations of the functions and structures of MCTs is necessary for the development of effective medicine which targeting these proteins. However, in comparison with MCT1, there is little information on location of the function moiety of MCT4 and which constituent amino acids govern the transport function of MCT4. The aim of the present work was to determine the molecular mechanism of L-lactate transport *via* hMCT4.

**Experimental approach:**

Transport of L-lactate *via* hMCT4 was determined by using hMCT4 cRNA-injected *Xenopus laevis* oocytes. hMCT4 mediated L-lactate uptake in oocytes was measured in the absence and presence of chemical modification agents and 4,4′-diisothiocyanostilbene-2,2′-disulphonate (DIDS). In addition, L-lactate uptake was measured by hMCT4 arginine mutants. Immunohistochemistry studies revealed the localization of hMCT4.

**Results:**

In hMCT4-expressing oocytes, treatment with phenylglyoxal (PGO), a compound specific for arginine residues, completely abolished the transport activity of hMCT4, although this abolishment was prevented by the presence of L-lactate. On the other hand, chemical modifications except for PGO treatment had no effect on the transport activity of hMCT4. The transporter has six conserved arginine residues, two in the transmembrane-spanning domains (TMDs) and four in the intracellular loops. In hMCT4-R278 mutants, the uptake of L-lactate is void of any transport activity without the alteration of hMCT4 localization.

**Conclusions:**

Our results suggest that Arg-278 in TMD8 is a critical residue involved in substrate, L-lactate recognition by hMCT4.

## Introduction

MCT4 (SLC16A3), is a member of the monocarboxylate transporter family [1], mediates transport of monocarboxylates, such as lactate, pyruvate and ketone bodies, across the plasma membrane [2]. The human isoform hMCT4 is composed of 465 amino acid residues and is predicted to contain 12 transmembrane-spanning domains (TMDs) with amino and carboxy termini facing the cytosol [1]. The transporter was characterized by heterologous expression in *Xenopus laevis* oocytes, exhibiting low affinities for most substrates and inhibitors compared to the affinities of MCT1 and MCT2 [2]–[8]. This protein is expressed strongly in glycolytic tissues such as white skeletal muscle fibers, astrocytes, white blood cells, chondrocytes and some mammalian cell lines [1], [6], [9]–[11]. Halestrap and Price reported that MCT4 might be of particular importance in organization that depends on high levels of glycolysis to comply their energy needs [12]. In fact, rat MCT4 is expressed in the neonatal heart, which is more glycolytic in energy metabolism than the adult heart in which MCT4 is absent [9], [13]. The protein is also expressed strongly in the placenta, which exports lactate, helping to maintain placental and fetal pH in times of glycolytic stress [14]. Furthermore, most solid tumors are known to rely on glycolysis for energy production and this activity leads to production of essential amounts of lactate. It is of interest that MCT4 is strongly expressed in some tumors [15]–[22]. A recent study showed that MCT inhibition had an important impact on tumor homeostasis [23], [24]. Additionally, silencing of MCT1 and 4 decreased cancer cell invasion and migration reduced glycolytic flux and tumor growth [25]–[27]. Hence, considering the role of MCTs in cancer, having an important effect on cancer cell viability, these transporters may be a very attractive therapeutic targets in cancer. We previously reported that hMCT4-mediated L-lactate transport is strongly inhibited by statins [28]. In addition, some MCT inhibitors were identified in other studies [27], . However, there has been little investigation of MCT inhibitors in a cancer context [27], [29]. Elucidation of the functions and structures of MCTs is necessary for the development of effective and fail-safe medicine targeting MCTs. However, compared to MCT1, there is little information on location of the functional moiety of MCT4 and which constituent amino acids govern the transport function of MCT4.

In order to verify the function of MCT4, we cloned and functionally expressed hMCT4 in *Xenopus laevis* oocytes to clarify the molecular mechanism of L-lactate transport in this expression system. We report here that a residue that is essential for substrate recognition by the hMCT4 transporter based on chemical modification and site-directed mutagenesis.

## Methods

### Amplification of the coding region of human MCT4

Total RNA was prepared from Caco-2 cells by using an RNeasy Product mini Kit (QIAGEN). The procedure followed the instructions provided. The cDNA gene of hMCT4 was obtained from RNA by using a reverse transcriptase (TaKaRa). BamHI and XbaI restriction sites were introduced near an initiation codon and a putative terminator, respectively. We designed primers based on the published sequence in GenBank™ database (accession number U81800). The sense primer was 5′-tagttcgcgactcgagggatccccgacgaaccaaccctcctggccatg-3′and the antisense primer was 5′-tagatcgcgagtcgactctagaccagctcagacacttgtttccggggt-3′. The underlined bases indicate the newly introduced restriction sites. The cDNA gene was amplified by the PCR method using the sense and antisense primers. This product (1.4 kbp) was subcloned into pGEM-T Easy vector. The construct was sequenced using a BigDye Terminator v3.1 Cycle Sequencing Kit® (Applied Biosystems).

### Site-directed mutagenesis

Oligonucleotides were custom-synthesized for the following sequences (Residues in **bold** are different from wild-type hMCT4.):

- R278-hMCT4 mutants forward:

5′-cattgacatcttcgcg**XXX**ccggccgcgggcttcg-3′,

where **XXX** was **AAG** for R278K or **CAG** for R278Q.

- R198-hMCT4 mutant forward:

5′-gccgcactcatg**XXX**cccctggtggtc-3′,

where **XXX** was **CAG** for R198Q.

Reverse primers for the hMCT4 mutant PCR reactions were the reverse complements of the forward primers. Site-directed hMCT4 mutants were obtained by using the Quikchange protocol (Stratagene).

### Expression of hMCT4 in Xenopus laevis oocytes

Capped cRNA from hMCT4 cDNA was synthesized using an in vitro transcription kit (Ambion). *Xenopus laevis* oocytes were collected under anesthesia (immersion in a solution of 1 g/l ethyl 3-aminobenzoate methanesulfonate salt) from frogs. Mature oocytes (stage IV or V) from *Xenopus laevis* were isolated by treatment with collagenase (1.0 mg/ml) for 60 min and then manually defolliculated. They were incubated in Barth's solution (88 mM NaCl, 1 mM KCl, 2.4 mM NaHCO_3_, 0.82 mM MgSO_4_, 0.33 mM Ca(NO_3_), 0.41 mM CaCl_2_, 10 mM HEPES, adjusted pH to 7.5 with NaOH) containing 50 mg/l gentamicin at 17°C overnight. On the next day, oocytes were injected with 50 ng cRNA in a 50 nl volume and incubated for 3–6 days. Oocytes injected with the same volume of water served as controls.

### Uptake Experiment in Xenopus laevis oocytes

The transport buffer used in this study was a standard buffer (100 mM NaCl, 2 mM KCl, 1 mM MgCl_2_, 1 mM CaCl_2_, 10 mM Goods buffer). HEPES was used for pH 8.0-7.0 buffer, MES was used for pH 6.5-5.5 buffer, and Homopipes was used for pH 4.5 buffer. Uptake of L-[^14^C] lactate in water-injected and cRNA-injected oocytes was performed at 25°C and oocytes were washed with ice-cold transport buffer. Thereafter, single oocytes were placed into scintillation vials and dissolved in 10% SDS. The radioactivity was determined by a liquid scintillation counter. The kinetic parameters *K*_m_ (Michaelis constant) and *V*_max_ (maximum uptake velocity) were calculated by fitting the data of the L-lactate uptake rate to Michaelis-Menten equation.

### Immunofluorescence confocal microscopy

The samples were sent to Sapporo General Pathology Laboratory Co., Ltd. Briefly, water-injected and cRNA-injected oocytes were fixed in 10% buffered formalin. The fixed oocytes were embedded in paraffin and stained with rabbit anti-MCT4 antibody (Santa Cruz Biotechnology). The samples were then visualized by using a confocal microscope (FV-10i; OLYMPUS).

### Materials

L-[^14^C] lactate was purchased from American Radiolabeled Chemicals. *Xenopus laevis* frogs were supplied by Hokudo. This study was approved by the Committee on Animal Experimentation, Hokkaido University. Standard chemicals and solvents were supplied by Sigma-Aldrich.

## Results

### Establishment of hMCT4-expressing oocytes

**Figure 1:**
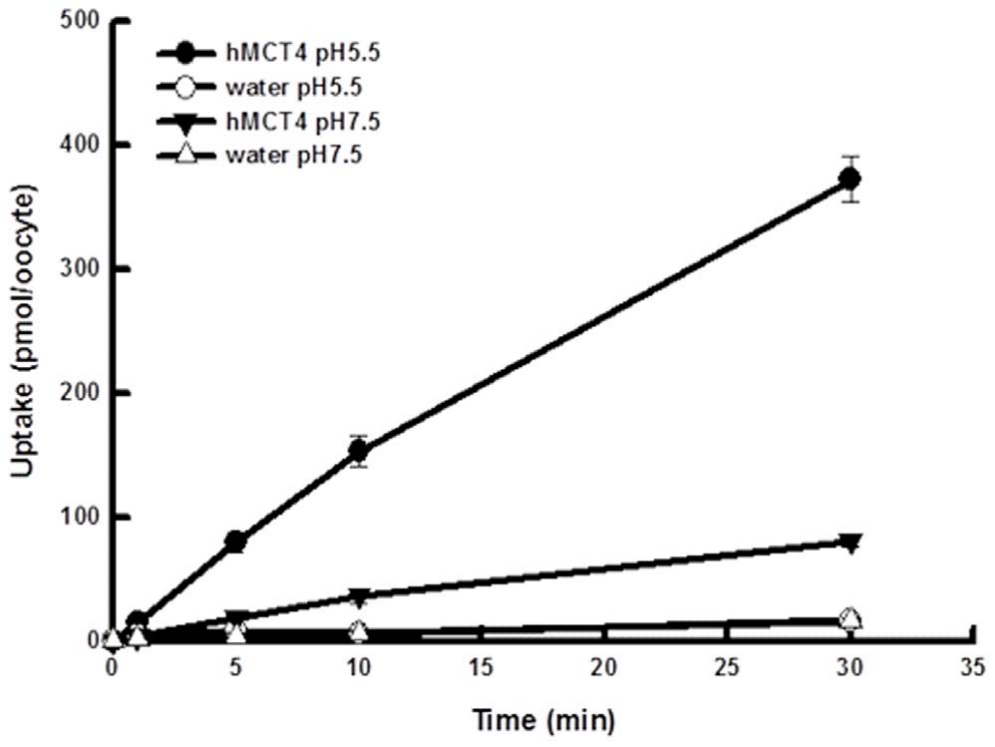
Accumulation of L-lactate in hMCT4-expressing oocytes. Oocytes were incubated for various periods at 25°C with transport buffer of pH 5.5 or pH 7.5 containing 0.1 mM L-lactate. Control oocytes were injected with the same volume of water instead of hMCT4 cRNA. Each point represents the mean ± S.E. of three – five experiments.

**Figure 2:**
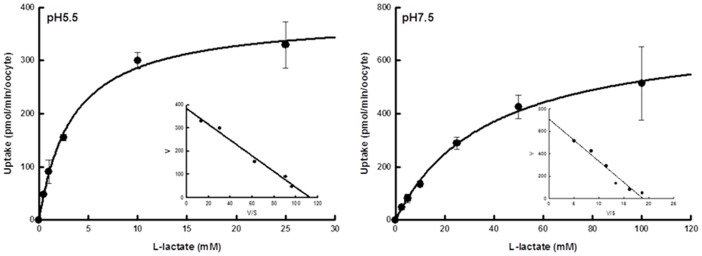
Saturation kinetics of hMCT4-mediated L-lactate transport. Uptake of L-lactate was measured with 10 min incubation at 25°C in transport buffer of pH 5.5 or pH 7.5 in the presence of an increasing concentration of L-lactate. hMCT4-specific uptake was calculated by subtracting the uptake in water-injected oocytes from the uptake in hMCT4 cRNA-injected oocytes. Only the hMCT4-specific uptake was used for kinetic analysis. The inset shows an Eadie-Hofstee plot of L-lactate transport activity. Each point represents the mean ± S.E. of three – five experiments.

The time course for the accumulation of L-lactate by hMCT4-expressing oocytes is shown in Fig. 1. The uptake of L-lactate was higher in cRNA-injected oocytes than in water-injected oocytes. The accumulation of L-lactate by hMCT4-expressing oocytes was markedly reduced by alkalizing the buffer pH, indicating that hMCT4 expressed in oocytes preserved the original characteristics of the proton-linked transport system. The accumulation of L-lactate was linear up to 10 min after incubation with L-lactate. Hence, the initial uptake rate was determined within 10 min after the onset. The relationship between initial uptake rate and L-lactate concentration is shown in Fig. 2, indicating that the uptake rate is saturated at high concentrations of L-lactate. The inset in Fig. 2 shows Eadie-Hofstee plots in which the line was straight, revealing that the uptake process is comprised of a saturable process.

### pH-dependency of L-lactate uptake by hMCT4-expressing oocytes

**Figure 3:**
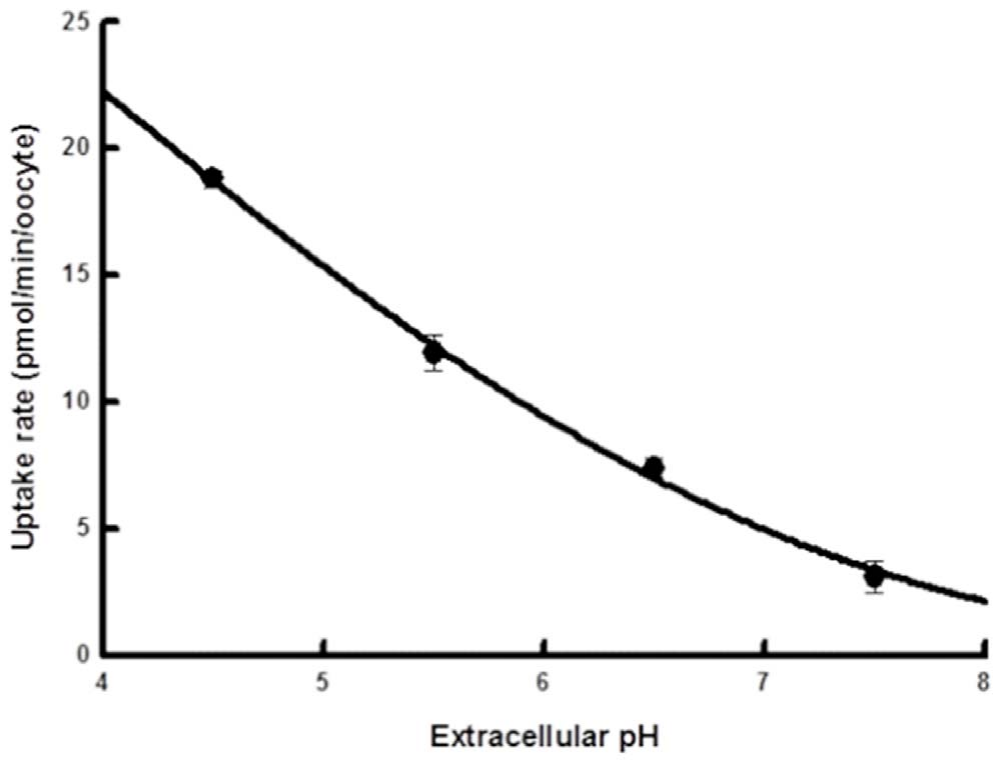
pH-dependency of hMCT4 activity in the presence of L-lactate. Uptake by oocytes was assayed for 10 min at 25°C in the presence of 0.1 mM L-lactate. Each point represents the mean ± S.E. of three – five experiments. The background uptake values of water-injected oocytes were subtracted.

The uptake of L-lactate by hMCT4-expressing oocytes was examined under various pH values from 7.5 to 4.5. As shown in Fig. 3, the uptake of L-lactate was decreased by alkalization of the extracellular pH, and the maximum values of uptake rate and values of the Michaelis constant showed a pH dependency (Fig. 2, Table 1). The kinetic parameters were calculated by fitting data to the equation. The *K*_m_ and *V*_max_ values were 3.4±0.4 mM and 383±17 pmol/min/oocyte at pH 5.5 and 37.6±4.1 mM and 710±54 pmol/min/oocyte at pH 7.5, respectively.

**Table 1:** Effect of extracellular pH on kinetic parameters of L-lactate uptake by hMCT4-expressing oocytes.

	Extracellular pH		
	5.5	7.5	- Fold (pH 5.5/pH 7.5)
*K*_m_ (mM)	3.4±0.4	37.6±4.1	0.09
*V*_max_ (pmol/min/oocyte)	383±17	710±54	0.54
*V*_max_/*K*_m_ (µl/hr/oocyte)	6.8	1.1	6.18

Data were taken from Fig. 2.

### Effects of DIDS and amino acid-modifying agents on the mechanism for L-lactate uptake

**Figure 4:**
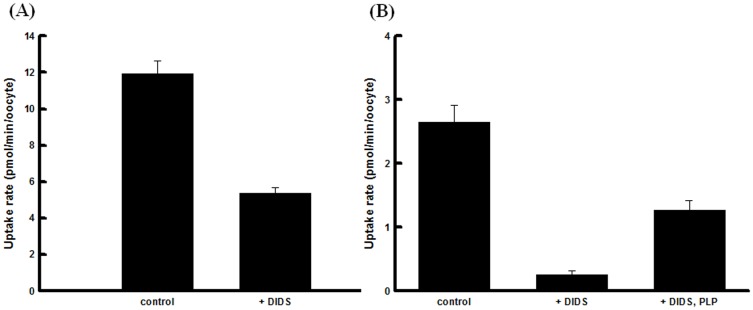
Effect of DIDS on transport activity *via* hMCT4. (A) Oocytes were incubated for 10 min at 25°C with transport buffer of pH 5.5 containing 0.1 mM L-lactate in the absence or presence of 0.5 mM DIDS. Data are presented as means ± S.E. of three independent experiments. The background uptake values of water-injected oocytes were subtracted. (B) Oocytes were preincubated at 25°C for 10 min with/without 5 mM PLP (pH 7.5). L-lactate uptake after preincubation was measured. The oocytes were incubated for 10 min at 25°C with transport buffer of pH 7.5 containing 0.1 mM L-lactate in the absence or presence of 0.5 mM DIDS. Data are presented as means ± S.E. of three independent experiments. The background uptake values of water-injected oocytes were subtracted.

**Figure 5:**
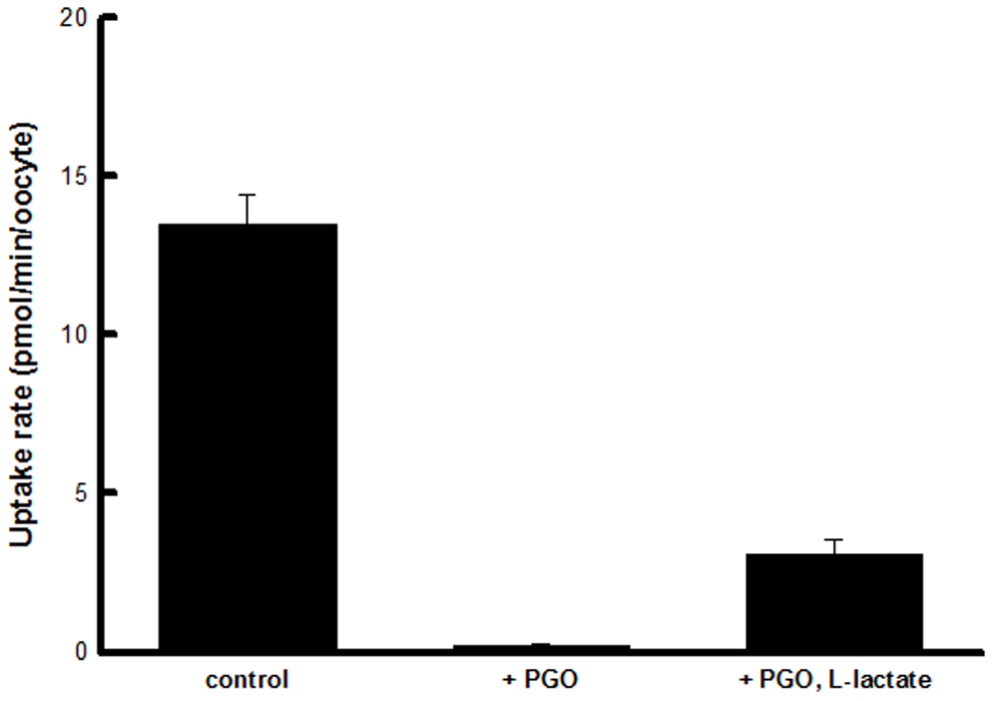
Effect of PGO modification on transport activity *via* hMCT4. Oocytes were preincubated at 25°C for 15 min with 100 mM PGO (pH 8.0) in the absence or presence of 1 M L-lactate. After treatment with PGO, oocytes were rinsed twice with transport buffer (pH 7.4). Oocytes were incubated additionally twice for 5 min with transport buffer. The oocyte were incubated for 10 min at 25°C with transport buffer of pH 5.5 containing 0.1 mM L-lactate. Data are presented as means ± S.E. of three independent experiments. The background uptake values of water-injected oocytes were subtracted.

Chemical modification is important for the study of a transporter structure-function relationship. The mode of action of amino acid-modifying agents as follows: 5,5′-dithio-bis(2-nitro-benzoic acid) (DTNB), thiol group modifier; dithiothreitol (DTT), disulfide modifier; 1-ethyl-3(3-dimethylaminopropyl) carbodiimide (EDC), carboxy group modifier; phenylglyoxal (PGO), guanidine group modifier; pyridoxal-5-phosphate (PLP), ε-amino group modifier; phenylmethylsulfonyl fluoride (PMSF), hydroxyl group modifier. It is well known that 4,4′-diisothiocyanostilbene-2,2′-disulphonate (DIDS) inhibits an anion transport by binding to the cell surface. As shown in Fig. 4, as in the case of other members of the MCT family, DIDS was found to be an inhibitor of L-lactate uptake. DIDS at a concentration of 0.5 mM reduced L-lactate transport to 44.8% of the control level at pH 5.5 (Fig. 4A). In the case of pH 7.5, L-lactate uptake decreased to 9.6% of the control level. This effect of DIDS was prevented by PLP, specific for lysine residues, modification (Fig. 4B). For further investigation to identify the amino acid residue(s) involved in substrate recognition, we examined the uptake activities under conditions of treatment with amino acid-modifying agents. Table 2 shows that, among the tested compounds specific for arginine residues, PGO showed the strongest inhibitory effect on 0.1 mM L-lactate uptake, which was reduced to 5.2% of the control level after 15 min of treatment. On the other hand, this abolishment was prevented by the presence of L-lactate (Fig. 5).

**Table 2:** Effects of amino acid-modifying agents on the transport system for L-lactate in hMCT4-expressing oocytes.

Modifying reagent (mM)	Selective for	L-lactate uptake
DTNB (1)	SH	98.5±6.7
DTT (10)	disulfide	89.8±12.4
PMSF (5)	OH	90.8±13.8
EDC (20)	COOH	103±11.3
PLP (5)	lysine	98.1±16.8
PGO (100)	arginine	5.2±0.5

Oocytes were preincubated at 25°C for 10 min with DTNB (pH 7.0), for 10 min with DTT (pH 7.5), for 10 min with PMSF (pH 7.4), for 30 min with EDC (pH 5.5), for 10 min with PLP (pH 7.5) or for 15 min with PGO (pH 8.0). L-lactate uptake was measured after treatment with the indicated concentrations of modifying agents. The oocytes were incubated for specified periods at 25°C with transport buffer containing 0.1 mM L-lactate. EDC with glycine methyl ether (GME)* and GME alone (data not shown) had no effect on L-lactate uptake. Data are expressed as a percentage of control in the absence of a modifying agent and are the means ± S.E. of three – five experiments.

* : GME was synthesized by esterification of methanol and glycine.

### L-lactate uptake in arginine mutants

**Figure 6:**
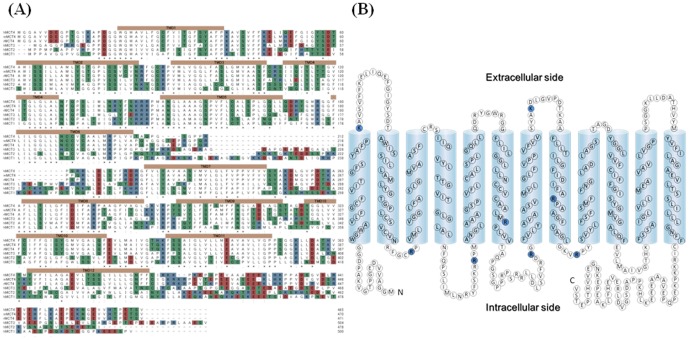
Sequence alignment and secondary structure of hMCT4. (A) Amino acid sequence alignment of human MCT4 with human MCT1, MCT2 and MCT3, rat MCT4 and mouse MCT4 transporter homologues using ClustalW. Orange bars in hMCT4 above the sequence are the regions predicted to from transmembrane-spanning domains (TMDs) by TMHMM. Polar residues are highlighted in red (acidic residues), blue (basic residues) and green (neutral residues). Identical residues are indicated by asterisks (*) represents under the residue. (B) Putative topology of hMCT4: The conserved basic residues of MCT1-4 are shown in blue.

**Figure 7:**
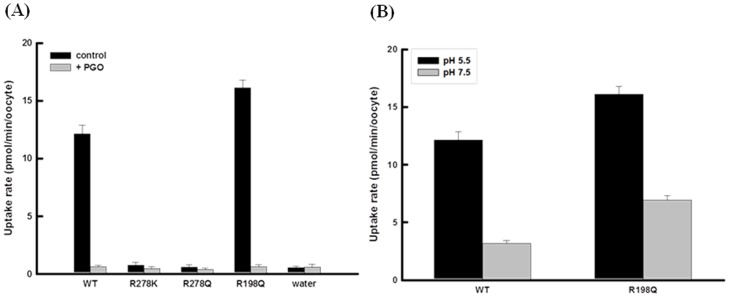
PGO effect and pH-dependency in hMCT4 mutants. (A) Uptake of 0.1 mM L-lactate was measured before and after treatment with 100 mM PGO in oocytes injected with hMCT4-WT or the indicated mutants. Data are presented as means ± S.E. of three – five experiments. (B) Uptake of L-lactate was measured in hMCT4-WT or -R198Q expressing oocytes with 10 min incubation in transport buffer of pH 5.5 or pH 7.5 in the presence of 0.1 mM L-lactate. Data are presented as means ± S.E. of four – five experiments.

The complete abolishment of transport activity of hMCT4 by PGO treatment indicated that an arginine residue plays an important role in L-lactate uptake. However, the position of the arginine residue is not known. To address this issue, based on a topology model of hMCT4 and multiple alignments, we mutated amino acid residues that might be involved in L-lactate transport. We refined this model by using alignment software such as ClustalW, matching the TM sequence signals predicted by TMHMM to TM-helices. There are 12 TMDs with the N-terminus and C-terminus located within the cytoplasm. The sequence alignment clearly showed that the greatest sequence conservation is in the putative TM regions and shorter loop regions between them. A pairwise basic local alignment search tool (BLAST) analysis showed that the amino acid identities relative to hMCT4 were 43% for hMCT1, 44% for hMCT2 and 55% for hMCT3. Figure 6 shows that hMCT4 has eight conserved basic residues, two lysine residues in the extracellular loops and four arginine residues in the intracellular loops. The arginine residue in TMD is conserved in many members of the monocarboxylate transporter family and we considered the two remaining arginine residues in TMDs, which were replaced with other amino acids, and the sequences of the resulting constructs were confirmed by DNA sequencing. Figure 7 shows L-lactate uptake and the effect of PGO in hMCT4-WT, R278K, R278Q and R198Q. hMCT4-R198Q induced L-lactate uptake comparable to that of the wild type and is PGO-sensitive and pH-dependency, although hMCT4- R278K and R278Q had no transport activity.

### hMCT4 localization in Xenopus laevis oocytes

**Figure 8:**
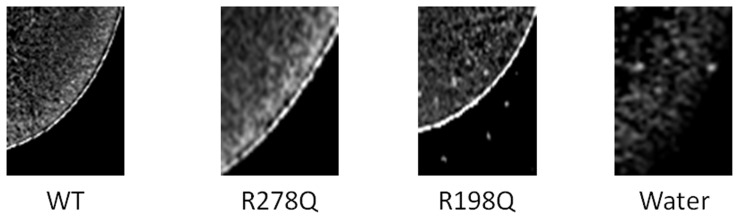
Fluorescence staining of MCT4 in slices of oocytes. Localization of hMCT4 in oocytes injected with hMCT4-WT or the indicated mutants. Oocytes were treated with antibodies against MCT4. Data shown are typical results of three independent experiments.

We performed immunohistochemistry to confirm the localization of hMCT4 in oocytes injected with hMCT4-WT or the indicated mutants. As shown in Fig. 8, staining of hMCT4-WT and the indicated mutants revealed a sharp signal for hMCT4 in the plasma membrane. On the other hand, no staining of hMCT4 was observed in water-injected oocytes.

## Discussion

We have shown dramatic attenuation of hMCT4-mediated L-lactate transport by amino acid modifier and point mutations. Structure and function analysis of hMCT4 followed by the use of a combination of site-directed mutagenesis and chemical modifications. In this study, we established hMCT4-expressing oocytes and kinetically examined the extracellular pH-dependency of uptake activity by hMCT4. Accumulation of the typical substrate L-lactate by hMCT4-expressing oocytes was more than twenty-times higher than that by water-injected oocytes. Transport activity was stimulated by a proton gradient. The *K*_m_ value was similar to a previously reported value for rat MCT4-expressing oocytes and *V*_max_ was larger than the previously reported value [6]. In contrast, another study showed that the *K*_m_ value was different [2]. The cause of this difference is 2′,7′-bis-(carboxyethyl)-5(6)-carboxy-fluorescein (BCECF) because this method is affected by pKa of monocarboxylate that accumulates in oocytes. Since hMCT4 is functionally expressed in the membrane, we established hMCT4-expressing oocytes for experiments on transport activity of hMCT4. Most solid tumors are known to resort to glycolysis for energy and lactate production. Lactate production leads to an acidic environment in cancer tissues [31]. Of interest, MCT4 is strongly expressed in some tumors. A recent study has shown that MCT inhibitors prevented invasion and metastasis of cancer, and MCT4 has been found to be an important poor prognosticator for patients with cancer [32]–[34]. Concentration-dependency of the rate of L-lactate uptake *via* hMCT4 was examined under pH 5.5 and 7.5 conditions. The results suggested that aberrant expression of proton-linked transporters could provide a selective advantage for cancer cells by providing lactate transport pathways that can exploit the acidic milieu associated with many solid tumors. The uptake of L-lactate was suppressed by alkalization of extracellular pH. These observations indicate that the abolishment of uptake at high pH may be due to disappearance of proton and/or substrate recognition by hMCT4, namely, it is thought that this is because the positive charge of the basic residue involved in proton and/or substrate recognition disappeared with alkalization. A simple way to explain the transport mechanism of hMCT4 is identification of an accessible amino acid residue in the substrate binding. This study demonstrated for the first time chemical modification of specific for amino acid residues on hMCT4-expressing oocytes. Identification of a functional amino acid residue by chemical modification is important for analysis of a protein structure-function relationship [35]–[43]. Chemical modifications except for PGO treatment had no effect on the transport activity of hMCT4, indicating that the amino acid residue involved in L-lactate transport mechanism is an arginine residue(s). DTNB had no effect, although *p*-chloromercuribenzene sulphonate (*p*CMBS) binding to a cysteine residue inhibits the activity of hMCT4 [2], indicating that the inhibition by *p*CMBS is caused by shutting out the substrate pathway for bulkiness of the inhibitor. We examined the role of the conserved lysine residue in extracellular loops using DIDS, an anion transport inhibitor, and using PLP, a lysine-selective modifying agent. hMCT4 exhibits less affinity than MCT1 for most inhibitors and has been reported to be insensitive to DIDS [2]. However, in this study, as in the case of the other members of the MCT family, transport activity *via* hMCT4 was markedly inhibited by DIDS and the potency of inhibition was changed by change in pH. On the other hand, this effect of DIDS was prevented by PLP modification, despite the fact that PLP alone had no effect. These results suggest that the lysine residue(s) in an extracellular loop(s), on a DIDS-binding site, may be extracellular pH-sensor, not a proton and substrate pathway. Therefore, in order to determine whether this arginine residue is involved in substrate recognition by hMCT4, we tried PGO modification, but the activity was completely abolished and investigation of the transport activity was therefore impossible. We fortunately found that modification in the presence of 1 M L-lactate preserved the transport activity, indicating that only the crucial arginine residue is protected by an excess of the substrate. At least, these data emphasize that an arginine residue located at or near the substrate-binding site is responsible for conferring substrate recognition. However, the position of the crucial arginine residue is not known. To address this issue, we mutated amino acid residue that might be involved in L-lactate transport. hMCT4 has six conserved arginine residues, two in the TMDs and four in the intracellular loops. In substrate uptake that is in an outward-facing state, it is very unlikely that arginine residues of intracellular loops participate in the substrate recognition. Thus, the two remaining arginine residues in TMDs, which were replaced with other amino acids. hMCT4-R198Q induces L-lactate uptake comparable to that the wild type and is PGO-sensitive and pH-dependency, although hMCT4-R278Q has no transport activity. In addition, substitution of R278 with lysine, so that the positive charge of the residue is conserved, showed that the arginine structure and not the charge is crucial for hMCT4 function. Our results indicate that no transport activity or PGO binding is detectable for Arg-278 mutant, which is also void of any transport activity. Complete impairment of the transport function in the Arg-278 mutant might be because the residue plays a critical role in activity of the transporter or it is required for correct localization of the protein in the plasma membrane. The assumptions concerned protein localization, are not correspond to the results of the immunostaining experiment. Arg-278 mutant expression level at the plasma membrane seems comparable to that of the wild type.

In conclusion, our findings clarified a substrate recognition site of hMCT4 by chemical modification and site-directed mutagenesis. The data regarding the L-lactate transport mechanism convince us that Arg-278 in TMD8 is the most probable residue involved in substrate recognition by hMCT4.
